# Notes on Medical Matters and Medical Men in Paris

**Published:** 1847-09

**Authors:** David W. Yandell

**Affiliations:** Louisville, Ky.


### WESTERN JOURNAL

OF

### MEDICINE AND SURGERY.

SEPTEMBER, 1847.

### Art. I.—*Notes on Medical Matters and Medical Men in Paris.* By David W. Yandell, M.D., of Louisville, Ky.

I take great pleasure in transmitting to you the following letter from Dr. Brooks, the young physician whom I have more than once mentioned as being engaged here in the study of Veterinary medicine. I am sure it will be read with interest. The Agricultural Society of Massachusetts is fortunate in having secured the services of an individual capable of devoting himself to the science with so much ardor as Dr. Brooks has manifested in the subject, and every merciful man will respond heartily to the wish so well expressed at the close of his letter, that the profession which holds a respectable position in all other civilized countries may soon find a place among the reputable avocations of our own. It cannot but strike one as strange that the diseases of the horse, an animal so much admired by Americans, and so necessary to the comfort and efficiency of our race, should have been so little studied. The odium which has hitherto been inseparable in our country from the term *horse doctor* will cease when the subject has enlisted such advocates as the author of this letter. I bespeak for it the careful perusal of the readers of the Journal.

*My Dear Yandell:*

The Veterinary Science, after many an age of the most unjust and inconceivable neglect, has at last received from the French Government a large portion of the favor and honor to which it is entitled. Especial colleges have been established at Lyons, Toulouse, and Alfort, in the northern, central, and southern points of the kingdom, whence numbers of intelligent young men, thoroughly grounded in the principles and practice of their profession, and imbued with those sentiments of self-respect and independence which true learning and unquestioned value never fail to impart, are yearly sent forth into society to fulfil, conscientiously, their honorable and important missions.

The "Horse Doctor," in the English sense of the term, has happily ceased to exist,—and if the medicine of man has not as yet extended a cordial hand to her younger sister, it is that *La Vétérinaire* has hardly had time to remove the enormous prejudices which ignorance and habit have accumulated against her, and that a certain feeling of hereditary dislike still lingers between the members of two sister sciences.

But the day is at hand, I hope, when the medicine and surgery of domestic animals shall be united, as in times of old, to that of humanity. The great principles of the healing art never change—their application alone varies. There should be then, but one *type* of medical schools, where the universal laws of science being imparted to all alike, their various subdivisions may by natural aid be perfected.

*Claude Bourgelat* is a name dear, for a thousand reasons, to the veterinary science. He alone foresaw its future utility and glory—the inestimable advantages which it is destined to render, and now renders, to medicine, agriculture, philanthropy—and only he was gifted with the energy and learning requisite to triumph over the numberless obstacles which obstructed his path. The labors of Bourgelat are almost incredible—naturally fond of the horse, he abandoned for the sake of this noble animal, a career in which he had just made his debut with the greatest credit, and gave himself wholly up to the study of his qualities, mechanism, and diseases. He revised the works of his predecessors, and exposed their many errors, and he has left to posterity some of the most remarkable treatises, upon nearly every branch of veterinary medicine, which have ever appeared. His opinion is still an authority, and even his faults bear the stamp of his honesty and of his genius. The name of *Claude Bourgelat, the Founder of the Veterinary Colleges of France*, is as familiar to the ears of the French students as are their own names, and his writings adorn the shelves of every well selected library.

But I almost forgot that I had promised to give you an account of the Royal College of Alfort. I will henceforth spare you all general reflections, and confine myself to my subject.

*Alfort* is, as you know, a little village situated upon the road to Lyons, about six miles from Paris. The veterinary school, to which it is indebted for its celebrity, contains upon an average some two hundred and fifty students, of whom about seventy-five enter annually, after having been previously subjected to a general, and, I think, insufficient examination. Diplomas authorizing the title of Veterinary Physician, are conferred upon forty-five or fifty; of this number nearly one-half are destined for the army—entering the several cavalry regiments with the title of *Under-Veteri-*

*nary-Assistants*, and with a rank corresponding to that of quartermaster.*

The duration of the studies is fixed at four years; examinations taking place at the close of each scholastic year, to decide whether or not the student is fitted to pass into a superior class, and to enter upon its more difficult pursuits. Such as fail to pass a satisfactory examination are obliged to recommence the studies of the year which has elapsed, and those that fail twice in the same examination, † are dismissed as wanting in capacity. As these periodical tests are pretty severe, there is always a certain number of *veterans* (as the backsliders are called) in the inferior classes. A student may thus, *à la rigueur*, be compelled to pass eight consecutive years in the college, and may even then fail to succeed in his final examination, and leave without a diploma.

Chemistry and physics, in their application to veterinary medicine, botany, pharmacy, anatomy, general and special therapeutics, surgery, and hygiéne are the principal branches of science taught at Alfort.

M. Eugene Renault, the Director of the institution, lectures upon Veterinary Jurisprudence, an intimate knowledge of which is all important to the practitioner.‡

The hospitals are spacious and well ventilated; they are capable of receiving nearly one hundred patients, at the moderate rate of fifty cents a day each for boarding, medical advice and surgical operations being entirely gratuitous.

Operations are performed either in the open air, or in the amphitheatre which is lighted from above, and in other respects adapted to the purpose. Their various stages and the accidents liable to attend them are thus advantageously seen by a large number of students at a time.

Diseases of the feet, as in the vicinity of all large cities,

---

* Maréchal des logis.

† Except in rare instances.

‡ I am about to give in a paper intended for a northern Journal, a sketch of the laws now in operation in France touching the question of *soundness*.

are very frequent at Alfort; and I think that—thanks to the admirable skill of M. Henri Bouley*—their nature and treatment are better understood here than elsewhere.

Each member of the superior classes has a patient entrusted to his care, for which he is held responsible, and for the state of which he is periodically called upon to account. There is a stable especially destined for abandoned and incurable subjects (*chevaux d'experience*), and within still another, situated at the extremity of the park, are confined glandered horses, and such as are affected with contagous diseases.

Vivisections, on an extensive scale, are performed by the students during the summer months; sixteen horses being sacrificed for this object weekly. Upon each animal are performed the capital and minor operations of veterinary surgery, such as lithotomy, extirpation of the fibro-cartilages of the ears, ligature of the carotid and other arteries, all operations on the feet, venesection in various parts of the body, extraction of the teeth,† etc. Eight students operate in turn upon each subject, when this is practicable; thus, about one hundred operations, of more or less importance, are performed upon each beast; as you may well imagine life rarely lingers to the last. I have no opinion to offer here upon this system, authorised by the French Government, of torturing animals to death, by way of recompense for the services which they may have previously rendered it. The sight is far from being a pleasant one, and its influence upon the minds of the pupils cannot, to say the least, be of a very elevating nature. Are the advantages of the practice sufficient to counterbalance its cruelties? Have we a right to cause one creature to suffer beyond measure, for the benefit of others of its kind—or for our own benefit? The inten-

---

* It is to be hoped that this gentleman will ere long publish, what is so much needed, a work upon Practical Veterinary Surgery.

† This cruel operation is not, I believe, enjoined by the regulations, but I have seen students extract molar teeth, *en passant*, for their own satisfaction.

tion of these vivisections is in part to habituate the student to the sight of blood, and to teach him to be ever upon his guard against unexpected efforts of his patient.

The museum contains some very interesting objects, that I have not space here to enumerate. The newly elected Professor of Anatomy, M. Gonbaux, will seize, I am sure, every opportunity of adding to its already valuable collection of anatomical and pathological preparations.

The dormitories are in a separate building, which also contains the refectory and kitchens. Each chamber is occupied by six young men. The bed-steads are of iron, (and here I may venture to suggest that it is to be regretted that their use is not more general with us than it is, as well for public schools as in hospitals). Each of its occupants is in turn bound to wash out and keep clean the common room.

There is a botanical garden attached to the institution, in one portion of which the most useful medical plants are cultivated, and in another, those only which are employed in Veterinary Pharmacy.

The dog-kennels are next to the garden; they are sufficiently extensive and pretty well managed. The patients are kept under shelter during the winter season, and in summer they are chained during the day time to the walls of the yard, where they receive the benefit of the air and sun. Affections of the skin, eyes and ears, tetanus, the distemper and hydrophobia, are among the disorders which I have oftenest observed among the dogs at Alfort. A series of very interesting and curious experiments were instituted last year, with the view of testing the contagion of this latter disease from the dog to other animals. I have seen it repeatedly produced in the horse and the sheep. There are few subjects more important and more interesting than this, and it is probable that we shall have before long from the able pen of M. Renault, the most perfect and learned treatise upon hydrophobia (or better still, *rage*) which has ever yet appeared.

There is always a *pig-stye* to be seen at the college, but it is rarely occupied by the animals for which it is intended

It is quite deserted at the moment in which I write. No very serious attention is paid to the maladies of the pig, although they are theoretically professed in the course of the session. The last occupants of the stye were of English race crossed with that of China. M. Viborg, the director of the Veterinary College of Copenhagen, has written an excellent treatise upon the diseases of this *edible* animal.

In their first year the students at the College at Alfort, are taught to forge, as also to form and apply shoes of various models to the foot of the horse. The forges are constructed upon simple and effective principles. There are six double furnaces at which the students work two at a time, in alphabetical order.

The dissecting rooms are in recently erected buildings, of which certain portions are yet unfinished. They consist of two spacious halls lighted from either side, and communicating with each other through the cabinet of the professor who, thus, is constantly close at hand with his scalpel and advice. For convenience of transport, the subjects are placed upon solid iron carriages, of a peculiar and convenient form.

The laboratory is to be removed at an early period to a new room expressly arranged for it. Another large building for the storing of fodder is in the course of construction.

A professor of horsemanship was formerly attached to the college, but for some reason or other the study of this accomplishment, so useful, if not essential, to the veterinary physician, above all if he be destined for the army, has been suppressed.

The drawing master has also disappeared. This, too, is to be regretted, for the art of drawing is of inestimable service to the anatomist and pathologist.

In this short letter upon the college of Alfort, I have not pretended to broach any of the numerous questions which at present agitate the veterinary world. I have perhaps written at once too little and too much, but should this superficial review fail to satisfy your interest and curiosity concerning the science, I may attempt, upon some future occasion, to do higher justice to so important a subject.

The Veterinary Colleges of France are the most perfect that exist; let us take them for our model, and seek to emancipate in the new world a noble science, that still struggles for independence and justice in the land of our fathers.

Your friend,

EDWARD BROOKS, Jr.

Alfort, June 8th, 1847.

*Medical Reform in France.*—A most important question, which has for some months past excited a large share of public attention, is the *Medical Reform Bill*, the discussion of which commenced in the Chamber of Peers on the 5th of June. Since any measure calculated to affect the interests of the medical profession in France cannot but be interesting to the profession in the United States, I have thought a brief analysis of the more prominent features of the bill, together with some notice of the existing laws in relation to the practice and teaching of medicine in France, might justly be considered as coming within the province of your correspondent.

Notwithstanding that France may be said to have taken the lead in medical reform for the last ten years, at least, it was not until the *Congrès Médical*, formed of delegates from among the medical practitioners of all parts of the kingdom, which assembled in Paris last November, to the number of about five thousand, that the numerous abuses were made known to the government, and assurances given by the Minister of Public Instruction that he would bring in at the earliest period a bill to relieve the wrongs against which the medical body had so long and perseveringly remonstrated.

Concerning the medical regulations established in March, 1803, to which additions and amendments have been made at different periods, by Royal ordinances and by the Council of Public Instruction, I will endeavor to give the leading points, avoiding, as much as possible, unnecessary details.

The medical body, as now constituted, consists of Doctors in Medicine, and *officiers de Santé;* the former graduates of one of the three universities of Paris, Strasburg, or Montpellier, and entitled to practice in any portion of France, while the latter, an inferior grade, are merely examined by medical juries and can only practice in the department in which this examination was passed.

In order to become a candidate for the former grade, M.D., the person must produce his act of birth; the consent of his father or guardian. if he be under twenty-one years of age; a certificate from a civil authority of good moral character, together with one or two minor requirements; and finally the diploma of Bachelor of Letters and Sciences, though the latter is dispensed with when the aspirant desires merely to become an *officier de Santé.* The period of study for the title of M.D. is four years, during which time the candidates take out sixteen inscriptions, as they are called, which are but certificates of attendance upon the prescribed courses, submit to five examinations, and defend a Thesis, at the cost of one thousand francs, and the price of the diploma being one hundred francs, the combined cost of the whole amounts to eleven hundred francs. The subjects of the examinations may be thus enumerated—

1*st Examination.*—Anatomy and physiology, dissection, the candidate being required to make some designated anatomical preparation in the dissecting rooms in six hours, relative to which he is asked questions.

2*d Examination.*—Internal and external Pathology with operations.

3*d Examination.*—Natural History, Physics, Chemistry, and Pharmacy, the candidate replying demonstratively to the questions addressed to him on chemical substances and medical plants.

4*th Examination.*—Medical Jurisprudence, Materia Medica, and Therapeutics.

5*th Examination*—Consists 1st, in a composition in Latin or French upon a medical or surgical question, the subject of which is determined by lot. 2d, in the examination of one or more patients in some one of the hospitals, after which they deliver their diagnosis and the treatment which they consider should be adopted. The thesis is required after this last examination. The subject is chosen by the candidate.

Thus, you perceive the first, second, and fifth examinations, and the latter part of the fourth, are eminently practical.

The *officiers de Santé*, as I have remarked, are not required to have the diploma of Bachelor of Letters; they undergo three oral examinations, the first, on Anatomy; the second, on the Elements of Medicine; and the third, on Surgery and Pharmacy. *Officiers de Santé* are prohibited from taking the title of doctor; though through a strange oversight in the law, they may with impunity assume the apellation of "*médecin*," given in common to doctors, *officiers de Santé*, and *vétérinaires*. The new bill proposes to remedy this. *Officiers de Santé* are not allowed to perform important surgical operations, except under the superintendence of a doctor of medicine. The penalties which may be at present enforced against persons practicing illegally, are: a fine of from one hundred to one thousand francs against any individual practicing as doctor; and a fine of from twenty-five to five hundred francs against those practicing as *officiers de santé*. In case of a second offence, the fine may be doubled, and the offender imprisoned for a period not exceeding six months.

This is the curriculum of medical studies for these two degrees, and the state of the medical profession under the present system.

In the new bill the leading points relate—

1st. To the two classes of practitioners, doctors of medicine and *officiers de santé*.

2d. To the repression of illegal practice.

3d. To foreign physicians who desire to practice in France.

The bill proposes the suppression of the *officiers de santé*, who are to be replaced by graduates in medicine who, for a fixed salary, are to give gratuitous medical attendance upon the poor. The Congress demanded this clause by an immense majority, and as Count Beugnot styled it in his report, it may be truly said to be '*la disposition capitale de la nouvelle loi.*' Relative to the second point, the illegal practice of medicine, the new law declares that any person practicing the healing art without having graduated in one of the French Faculties, or without a duly legalized authorization from the French Government, shall be liable to imprisonment for a period of not less than six months, and not exceeding two years; for the second, imprisonment, the minimum period of which is two years, and the maximum five years. A clause at first inserted in the bill, but which has been modified by the committee, provided that all medical men, who might incur the slightest punishment of the simple correctional police, should be deprived of their right to practice. This, as you may well suppose, excited almost universal disapprobation, and certainly, knowing as every Frenchman does for how very trivial offences one may be punished by that not always perfectly just tribunal, the correctional police, not without reason. On the third point, namely, the practice of foreign medical men, it is proposed by the new law that no foreign physician shall be authorized to practice in France, unless it shall have been previously decided by the Royal Council of Public Instruction, that his diploma is equivalent, as an attestation of length of studies and respectability of the university which conferred it, to that granted by the French Faculties. Further, the authorization may be restricted to a certain locality, and confined to a limited period, and is always revocable at pleasure. They are amenable in the same extent as French practitioners to the present laws concerning punishments, and will be in the same degree to any that may be hereafter established. Concerning foreigners who desire to take the degree of Doctor of Medicine in the French Faculties, the diploma of Bachelor of Letters of some university, whose degrees are considered equivalent to those of France, is required. And doctors in medicine or surgery, graduates of foreign faculties who desire to obtain the same grade in one of the faculties of

France, of which, as I have before remarked, there are three, are required to undergo all the trials of the *doctorate*, that is to say, the five examinations and the thesis. They must previously address a request to the Minister of Public Instruction in order to obtain the inscriptions, which are allowed in the proportion of two-thirds of the time spent in foreign universities. Thus, to obtain the sixteen inscriptions equivalent to the four years of study necessary for the doctorate, he must show by certificates that he has studied six years in these universities. The price of the diploma is the same as though he were an inhabitant of France, that is one hundred francs.

The Faculties of France may be said to consist 1st, of Professors of the Faculties of Medicine who lecture on the various branches of medical science; 2d, agrégés or assistant professors; and 3d, of *professeurs particuliers*, or private medical teachers. Professors, assistant professors, and private teachers are all nominated by *concours*. According to the present system doctors in medicine are allowed to contend for any vacancy that may occur in the professorships, and the consequence is that *concours* are incessantly going on, and the host of competitors is often very disproportioned to the importance of the places sought. Thus, last year, there were two vacancies for the situation of surgeon to the hospitals at Paris. There were thirty-two candidates, and the concours lasted five months. The new law proposes to allow only agrégés to be eligible.

Notwithstanding the herculean labors of the medical profession of France in the field of pathological anatomy, while they have laid the medical body throughout the world under lasting obligations and given them an enviable and just celebrity, attracting pupils from every quarter of the civilized globe; notwithstanding the magnificent bequest of Dupuytren of $40,000, for the establishment of a chair of morbid anatomy, a bequest, the spirit of which has been so zealously carried out by the indefatigable and earnest M. Orfila, there is much yet remaining to be done. The new law acting upon this principle provides for the formation of laboratories, in the faculties and secondary schools, where the student will be forced, by frequent *post-mortem* examinations, to acquire that knowledge of organic lesions which is now deemed so essential a part of the education of the intelligent and accomplished physician.

I append an account of medical education in Great Britain which I met with in a late English Journal, and during my tour through Germany shall endeavor to afford a similar notice of the profession in that country, thus furnishing to the readers of the Journal an outline of the laws regulating medicine in the three nations in which it has received its highest cultivation.

"In Great Britain, the degrees granted to entitle persons to practice medicine are those of doctor of medicine and of licentiate of the college of surgeons; the latter, indeed, should, strictly, only confer the privilege of practicing surgery; but in England, especially, we find a numerous body of practitioners, who have no *medical* degree, yet practice every branch of the profession. The degree of doctor of medicine is conferred in England, by the Universites of Oxford, Cambridge, and London; in Scotland, by those of Edinburgh, Glasgow, St. Andrew's, and Aberdeen; in Ireland, by the University of Dublin, and by the College of Physicians. The surgical degree is conferred by the college of surgeons in London, Dublin, Edinburgh, and Glasgow. The medical practitioners thus constituted are of four classes—pure physicians, pure surgeons, (these two are common to the three kingdoms); physicians who are also surgeons (peculiar to Scotland and Ireland); and general practitioners, a class peculiar to England, who only take out the degree of surgeon, and become members of the Apothecaries' Hall in London. They engage in the practice both of medicine and surgery, and in addition dispense their own medicines. They constitute by far the largest medical body in the United Kingdom, and have of late years formed a powerful association, the object of which is to obtain from government the privilege of founding a college where degrees will be conferred. This was chiefly brought about by the partiality exhibited by the college of surgeons in its nomination of fellows, the practitioners being in a great measure excluded. Persons not legally qualified to practice are subject to prosecution by the various colleges; the President of the College of Physicians in London having the power of issuing his warrant to bring before him any person illegally practicing within his jurisdiction.

"The schools of *medical instruction* in the three kingdoms are of two kinds—Universities and Colleges, established by royal charter, the professors in which are elected by the General Council; and, secondly, private schools, self-constituted, with self-elected professors. Here we may at once perceive a grave defect, in the permitting men unknown, untried, to instruct others, when they themselves may be grossly ignorant. Thus young men are, perhaps, for years learning what they will have to forget and unlearn afterwards; whilst, should they have not sense to discern, or energy to commence anew, they are thrown upon the public, imbued with false ideas, skilled in *deadly* practice. Such evils, if not entirely prevented, are at least in a great measure avoided where professors are chosen by a public body, the reputation and standing of that body depending to a certain degree on the character and acquirements of the professors it selects; its pupils being drawn to it by their confidence in such selection; whilst the private schools being in many instances but joint-stock associations for putting money into the pockets of self-created teachers, are flocked to by students, as places where they may take out the necessary certificates in the easiest and cheapest manner. For, strange to say, the prices of the various lectures, as well as the length of time required to be spent in professional study, vary in these different establishments; thus we may at any moment see, in the London newspapers, advertisements from different schools and hospitals, literally underselling each other. Certain Universities, as those of Dublin, Edinburgh, Oxford, and Cambridge, will not receive the certificates of lecturers teaching in Colleges of Surgeons, even when teaching surgery; whilst, on the other hand, the College of Surgeons in Dublin retaliates, by refusing the certificates of lecturers in these Universities; though it frequently occurs that the very same individual, who is one year professor at the College of Surgeons, is the next seated in the Academic chair of the University. Again, the Universities of Dublin, Oxford, and Cambridge will only confer the degree of Doctor of Medicine on persons having graduated in Arts, and then only in about nine years after first entering the University; whilst the Scotch Universities and the Colleges of Surgeons give their degrees without requiring any such graduation in Arts, some after three, some after four, some after five years of study. Yet, with such a difference in the length of time in the course of study required, the general privileges and the titles conferred are the same. Here again, however, a strange anomaly appears; for in certain cities there are privileged bodies, whose members alone are by law entitled to practice in those cities, or within a certain limit of their boundaries. Thus, though the London University confers the degree of Doctor of Medicine, and such Doctor of Medicine is considered fully competent to practice his profession generally throughout the United Kingdom; yet, he dare not, *legally*, practice in London, or within a range of seven miles. Doctors of Medicine of the Scotch Universities, again, are not by law entitled to practice in England, nor in Dublin, except they have the license of the Dublin College of Physicians. And what is the necessary consequence of all this want of uniformity in the regulation of medical practice? Why, that the difficulties thus thrown in the way of recognising who has, and who has not, a right legally to exercise his profession, afford a fair field, and open a broad gate to gross humbug and impudent quackery. And so we find, that in no one country on the surface of the globe has quackery ever reached to such a height, or humbug elevated itself on so lofty a pinnacle, as in England. It matters not of what kind, or what description, so it be but quackery. St. John Long with his liniment—Animal Magnetism, with its somnambulists—Homœopathy, with its millionth-fraction doses—Hydropathy, with its water and its blankets—Holloway, with his ointment—all! all alike have gulled, alike have robbed, alike have killed the British public. Why, it has been calculated, from undeniable statistics, that the Battle of Waterloo was fatal to far fewer numbers than the pills of one advertisement of a celebrated quack within a short period of years. Yes, in England quackery sits enthroned on a hecatomb of fated dupes, because the English Legislature will not interfere, and, by equalizing instruction, and conferring similar rights on similarly educated men, cause the public to rely on their skill, and place confidence in their attainments. Science itself is depreciated, and men of science inevitably lowered in public estimation, in any country where the pretender is permitted to enjoy the same privileges and rights as the true man; whilst protracted misery, incurable disease, and agonizing death must fall to the lot of thousands thus allowed by a careless Legislature to be yearly sacrificed to ignorance and quackery."

Your readers know the celebrity of Ricord as a writer on venereal diseases. I translate the following case from his work just published on that subject:

*Case of Syphilis.* R. J., 31 years of age, born in Ireland, was admitted into the hospital *Du Midi* on the 23d of June, 1846. He was born of healthy parents and possessed a good constitution, of lymphatic temperament. He had had in infancy a slight attack of small pox.

He contracted, nine years ago, a gonorrhea, and at the same time a chancre upon the glans penis, which was soon accompanied by two bubos. The bubos took on an acute inflammation, *suppurated*, and having been opened by the aid of a bistoury, remained unhealed for more than a month afterwards. The chancre was not cicatrized until the end of four months. During the whole of these accidents an energetic mercurial treatment was practiced, which caused a severe ptyalism. No other symptom supervened, and his health remained perfect during the four following years. At this period, R***, who had still continued to see women, observed a new swelling in one of the inguinal regions without other antecedent; the swelling was of an indolent character; little inflammation or pain existed; nevertheless it attained considerable size, and terminated in suppuration. Seven weeks expired after the spontaneous opening of this bubo before it healed. This time no specific treatment was employed, and for three years no accident supervened. Two years since, a few days after connection with a public woman, the patient perceived a little excoriation in the fossæ of the glans penis; this excoriation suppurated slightly and was accompanied by very little itching. It soon presented a rounded appearance; its surface was of a grey color, and its base, which had gradually attained the size of the little finger nail, became prominent and excessively hard. Scarcely fifteen days had elapsed since the commencement of this indolent ulceration, when already several glands of both the inguinal regions became swollen, though the tumefaction was not very great, and was unattended by any pain. The patient was submitted to a pretty energetic mercurial treatment for four months, at the same time he took bitter infusions and put himself upon a severe regimen. The chancre healed slowly, and the swellings in the inguinal regions disappeared *without suppurating*. Notwithstanding the mercurial treatment, however, ill defined pains came on during the third month, in the neighborhood of the great articulations, with a supra-orbital headache at night, both of which disappeared at the end of a month,—the patient was thought cured and the use of mercurials was suspended. Eight months later, however, some ulcerations appeared on the tonsils, the posterior cervical glands became swollen, and there was some difficulty in the movements of the neck. It was deemed necessary to have recourse again to mercurials, and continue their use for nearly three months, when the cure was considered radical and their employment relinquished.

Six months since the patient noticed upon his legs little spots of a dull red color, unaccompanied with pain or itching. These spots were succeeded by little suppurating pimples which gradually increased in size, became more and more purulent, and finally terminated in a scab. This scab at first of little extent, slowly increased in size by its circumference, while in proportion its centre became convex. At its points, when the increase was still going on, an areola of a claret red, at the same time surrounded and preceded each new zone of the epidermis, which the more recent suppuration had elevated, and which, drying in its turn, served to extend still further and further the base of the scab. This, after being detached, gave vent to a thin, reddish, sanguinolent pus. In the points where the eruption had ceased to make further progress, the scabs, at first of a yellowish, then of a brownish green, became black; they were hard like horn, giving upon percussion a dry sound; presenting no longer the appearance of an areola, but were surrounded by a narrow circle where the epidermis was in a state of degeneration. Finally, the eruption was limited to the lower limbs, where there were four scabs upon the right and one upon the left. Besides, upon the right, the elementary point of departure was still to be seen what consisted only in a vesicular elevation of the epidermis, with an extensive areola of a dull red color. This form of eruption which always commences with a small vesicle and not by a bulla, can be arrested at this period, or it may pass through a succession of developments which class it often with ecthyma, before arriving at those proportions to which the epithet of *rupia* can be applied. However this may be, after the fall of the scabs which had been hastened by the application of poultices, beneath the one which occupied the region below the patella an irregular ulcerating surface was observed, which from its appearance indicated that it had served as the base to a cluster of eruptions. A part of its surface was already cicatrized. In other points the ulcerations being united, of an irregular round form, presented jagged inverted edges, while their bases, of a brownish green color, were covered with a pultaceous matter similar to that observed in primitive accidents. The scabby crust upon the anterior part of the leg concealed a surface of vermilion red, which was formed of fleshy granulations of a healthy character, somewhat elevated above the level of the neighboring parts. In other places, the crusts or scabs concealed only radiated cicatrices of a brown color, already somewhat contracted and presenting a vascular arborescence. The eruption which dated now six months, was not the seat of any itching, and only became painful when the patient was standing or walking, which caused the scab to crack, which was followed by suppuration and sometimes bleeding. Six weeks before the entry of the patient into the hospital, and a few days after his last sexual connection, an ulceration which attacked a part of the glans was developed upon the semi-mucous reflection of the prepuce. This ulceration assumed the pultaceous phagadenic form.

The treatment consisted in the use of a decoction of *quassia amara* sweetened with the syrup of gentian, and during the first eight days a pill composed of one grain of the protoiodide of mercury and three grains of the iodide of potassium; eight days after the dose was increased to two pills, then three, then four, each at intervals of eight days. The iodide of potassium was given in the dose of three grammes (about ʒ2 gr.14) during the whole course of the treatment. After the crusts became detached the ulcerations were dressed with a solution of the tincture of iodine (2 grammes of the tincture of iodine to a hundred grammes of distilled water, with a sufficient quantity of the iodide of potassium to dissolve the iodine).

After six weeks of treatment the ulceration upon the penis was healed, but it was not until the end of the fourth month that the legs were entirely so. Six months later the cicatrices were still a little bluish at their centres, of a dull white at their circumference, and presented a radiated honey-comb appearance. They resembled the scars produced by burns.

The case that we have just presented to the reader, is interesting under more than one point of view, and constitutes in itself one of those cases which illustrate the whole doctrine. The patient contracts, in the first instance, a gonorrhea, a chancre and some bubos; he detects no *induration* of the base of the chancre; the bubos *suppurate;* no constitutional symptoms supervene; it is true a mercurial treatment was pursued, but we shall see further on if it is to this circumstance that the absence of all consecutive symptoms should be attributed. A long time after this first affection, and a few days after suspicious sexual intercourse, a bubo makes its appearance, a bubo without *any other antecedent*, and which the supporters of the doctrine that bubos may exist as primary symptoms should class in this category. The swelling of the glands—of which the course has evidently been that of a subacute strumous inflammation or of a sympathetic idiopathic glandular affection, suppurates,—no specific treatment is employed, and no secondary accidents occur. Finally, a new ulceration of the genital organs follows another intercourse, which according to the patient's account becomes extremely hard. The swelling of the glands which accompanies it does not become voluminous, remains indolent, and does not suppurate. The mercurial treatment is begun afresh, and like the first lasts four months, but nevertheless does not prevent the successive evolution of constitutional symptoms for the reason that it is inefficient. After the last intercourse a new ulceration appears upon the penis—now that the syphilitic diathesis is already formed; it takes on the *phagadenic form*, the patient having lost his predisposition to contract an indurated chancre. This is what we witness every day when we observe well, when we rigorously follow the chain of symptoms and the connection of cause and effect. Let us learn then to appreciate the nature of these manifestations. Let the physician who collects cases for the benefit of science take the trouble to write their history himself. Let him sketch the features of the disease and give its diagnosis without permitting himself to be deceived by the prejudices or ignorance of the patient. Then when the diagnosis has been accurate, when account has been taken of all the causes of error that we have pointed out elsewhere, we can affirm that no constitutional symptoms will follow as a sequel to a non-indurated chancre with a suppurating bubo. The diathesis does not become established, and consequently a mercurial treatment, to which we might be disposed to attribute the honor of the cure, is not only useless but may be injurious, may impair the constitution and tend to render, as the foregoing case seems to prove, the ulterior accidents more serious. We can also rest assured that an inflammation of the glands which does not depend upon indurated chancre will not (whatever may be the treatment) be followed by a constitutional infection. But we can affirm that when a chancre presents the specific induration, when the swellings of the neighboring glands remain indolent and do not suppurate, that the diathesis is established; that in a given time and within certain determinate limits the appearance of constitutional symptoms will be inevitable; that the mercurial treatment given at the proper time, form, and dose, may, perhaps, entirely prevent their appearance or only retard or arrest it in some of its manifestations, as the present case exemplifies.

Finally, when the diathesis is established it does not appear more disposed to multiply itself than other diatheses; if the patient contracts new primitive symptoms these remain local and, as a proof this disposition, they no longer become indurated. But on the contrary, if the constitution has been a long time under the influence of the sympathetic diathesis, which has not been subjected to a methodical treatment, or if a bad treatment, or especially a mercurial treatment has im paired the constitution, then the primitive symptoms have a great tendency to take on a severe type, and often one of the varieties of the phagadenic form.

The surgeon of *La Pitié* is no more! The iron-framed, the strong-minded, the fearless Lisfranc is gone. Whatever may have been the opinions of his professional associates respecting his heart, no one can deny that he has left ineffaceable marks of his intellect upon the vast fields in which he labored; and although but few of them followed his remains to their final resting place, the time will come when, viewing his career with less of prejudice and animosity, all will pronounce upon him a fair and just judgment and number him among the "law givers" of medicine.

Jacques Lisfranc was born in the department of Loire in the year 1789. Commencing his studies at Lyons, he received the degree of Doctor of Medicine in Paris in 1812. He entered the campaign of Dresden as assistant physician to one of the divisions of the army, and was honorably noticed by its great commander. Upon his return he filled the position of assistant physician at *Hôtel Dieu* in Paris. In

1818 he entered with success the concours for the *bureau central;* in 1823 he was equally successful in his contest for the assistant professorship in the faculty; in the following year he was appointed second surgeon to *La Pitié* hospital, and almost immediately afterwards, Béclard dying, he became first surgeon.

It was here, and dating from this period, that he began to make his investigations concerning the diseases of the genital organs of the female, and it was also about this time that he turned his mind to operative medicine, for which he has since done so much.

The mind of Lisfranc cannot be said to have been a quick or brilliant one, but it was patient, acute, and laborious, a mind for analysis and minute investigation, that, imbued with a sound erudition, moved with regular and collected steps. Hence the vigor and perspicuity with which he describes his operations. Hence the value of his clinical lectures, and it is on this account, as much as on any other, that the medical world has reason to deplore that death did not spare him until at least he could have completed the two works on which he was engaged, *Médicine Operatoire*, and *Clinique de l'hôpital de la Pitié*. Few writers have published more extensively than Lisfranc. Commencing with his inaugural thesis, he has continued, in the midst of his onerous hospital duties, to which he attended with a conscientiousness well worthy of imitation, and in the midst of one of the largest private practices in Paris, to bring forth memoir upon memoir with a rapidity truly surprising. The works upon which he was laboring at the time of his death were meant by him to be his most important, and his chief regret seemed to be that he could not live to complete them. Since his inaugural thesis, which he published in 1813, he has given to the world memoirs on the following subjects, besides others that I shall not stop to enumerate: Upon the amputation of the arm at the shoulder joint; upon the amputation of the foot at the tarso-metatarsal articulation (1815); upon a new method of operating for stone in the female; new methods and processes in amputating at the scapulo-humeral and coxo-femoral articulations (1823); upon the employment of the chloro-oxide of sodium and calcium in the treatment of simple ulcers; upon amputations practiced upon the lardaceous and non-scirrhous tumors; upon lachrymal tumors and fistula cured without operation (1826); upon white swelling of the articulations; upon scirrhus; upon the general rules for disarticulations; upon compression employed to reduce indurations.

As a teacher Lisfranc was much admired, and his clinics were attended by crowds of pupils anxious to listen to his clear, practical instructions, and witness the operations in which he exhibited so much skill. His personal appearance aided in raising him to an eminence which his superior intellect enabled him to maintain. He was lofty in stature, of a stately gait, and robust form; his features though large were regular, and his deep-set, restless, penetrating eye, expansive forehead, and pale, anxious, thoughtful expression, gave to him an appearance in which the beholder was at once interested. His temper was an unhappy one, owing in part, it is said, to disappointment, and this infirmity kept him in incessant feuds with his professional brethren.

During the life time of Dupuytren he was the object of Lisfranc's peculiar hatred, and after the death of that great rival, Velpeau became the subject of his bitter invectives. In the midst of one of his most interesting lectures, without any apparent provocation, not unfrequently he would pause, drop his subject, and pour forth a furious philippic against some colleague whose doctrines or behavior had excited his displeasure. It is not to be denied that his manner to his patients was also harsh and impatient, and that in his morning visits he sometimes gave himself up to sudden transports of anger. Pupils, hospital physicians and patients were alike required to yield perfect and entire submission, not only to his opinions, but his whims and caprices, any departure being visited, instantly and in public, by sharp rebuke garnished with curses. Still, Lisfranc was possessed of generous qualities of the heart for which he has not had credit. He was capable of magnanimity towards his rivals and revilers, as the following anecdote will testify. One of these, who had been exceedingly bitter against him about the time that he promulgated the idea of amputating the neck of the uterus, became a candidate for a vacancy in the Royal Academy of Medicine. Lisfranc was the first to second his claims and to press his merits upon the attention of the members. By his vote and his influence the candidate was raised to the eminence he coveted. When some one afterwards expressed to Lisfranc surprise that he should have aided one known to be so much his enemy, he replied that "where talent and science were concerned he always voted with his head, never with his heart."

Instances of this kind are not rare in Lisfranc's history, and although in the course of his long, eventful life he made many bitter enemies, he has left behind him many true friends. His purse and his professional services were ever subject to the calls of charity, and in his death many a poor family has lost a stay and support, a kind and ready benefactor. The church on the day of his burial was filled with the poor to whom he had rendered services, and many were the tears that trickled down the tanned faces of the laboring men as they took their places in that long funeral procession.

He died of pseudo-membranous croup, after four weeks of suffering. He requested that tracheotomy might be performed, but the operation was declined, as false membranes were already formed in the pharynx, œsophagus, larynx, bronchi, &c.

In the course of the few short months of this year how many lights have gone out in our profession! Warner, Revere, McClellan, Wagner, Ramsbotham, Lisfranc—"how fast has brother followed brother from sunshine to the sunless land!" Peace to their manes, honor to their dust!

Paris, June 30th, 1847.

